# Salivary Microbiome in Pediatric and Adult Celiac Disease

**DOI:** 10.3389/fcimb.2021.625162

**Published:** 2021-02-17

**Authors:** Dimitri Poddighe, Almagul Kushugulova

**Affiliations:** ^1^ Department of Medicine, Nazarbayev University School of Medicine (NUSOM), Nur-Sultan, Kazakhstan; ^2^ Department of Pediatrics, National Research Center for Mother and Child Health, University Medical Center, Nur-Sultan, Kazakhstan; ^3^ Laboratory of Human Microbiome and Longevity, Center for Life Sciences, National Laboratory Astana, Nazarbayev University, Nur-Sultan, Kazakhstan

**Keywords:** celiac disease, salivary microbiota, microbiome, children, adults

## Abstract

The human salivary microbiota includes hundreds of bacterial species. Alterations in gut microbiota have been explored in Celiac Disease (CD), but fewer studies investigated the characteristics of salivary microbiome in these patients, despite the potential implications in its pathogenesis. Indeed, some recent studies suggested that the partial digestion of gluten proteins by some bacteria may affect the array of gluten peptides reaching the gut and the way by which those are presented to the intestinal immune system. The available clinical studies investigating the salivary microbiota in children and adults, are insufficient to make any reliable conclusion, even though some bacterial species/phyla differences have been reported between celiac patients and controls. However, the salivary microbiome could correlate better with the duodenal microbiota, than the fecal one. Therefore, further clinical studies on salivary microbiome by different and independent research groups and including different populations, are advisable in order to explore the usefulness of the salivary microbiome analysis and understand some aspects of CD pathogenesis with potential clinical and practical implications.

## Introduction

The microbial communities naturally colonizing the gastrointestinal (GI) tract are an essential and physiological component of the healthy human body, through a symbiotic interaction, whereby the former player contributes to several metabolic, physiologic, inflammatory, and immunologic functions for the latter one. In detail, the microbiota plays a role in regulating the host immunity by influencing the development and homeostasis of the gut epithelial layer and mucosal-associated lymphoid tissue (Nardone et al., 2017; Dieterich et al., 2018).

Alterations in gut microbiota (or dysbiosis) have been explored and investigated in several GI diseases, including Celiac Disease (CD) (Collado et al., 2007; Nadal et al., 2007; Schippa et al., 2010). CD is a systemic immune-mediated disease, characterized by a variable pattern of GI and extra-GI clinical manifestations, but it is clearly defined by the presence of a gluten-dependent atrophic (small bowel) enteropathy along with a consistent serological panel (positivity for anti-tissue transglutaminase antibody and/or anti-endomysium antibody) (Lebwohl et al., 2018; Lindfors et al., 2019).

The dietary intake of gluten, known to be a necessary trigger for the development of CD, and the predisposing HLA-DQ2 and/or HLA-DQ8 genotypes (virtually carried by all CD patients), cannot completely explain alone the disease pathogenesis. Therefore, among several hypothetical and additional etiologic factors, a role for the gastrointestinal microbiota in the switch from the immunological tolerance to the pathologic immune response to gluten, has been considered in CD patients and especially in children (Olivares et al., 2014; Olivares et al., 2018a; Rintala et al., 2018).

Each GI tract is characterized by a peculiar microbiota, in terms of qualitative and quantitative bacterial composition. In general, the bacterial density progressively increases along the GI tract, ranging from 10^3^ colony-forming units (CFU) per gram (g) of luminal content in the duodenum up to values of 10^12^ order in the colon; concomitantly, also the bacterial diversity (in terms of number of different bacterial species) gradually increases from the proximal to the distal GI parts (Dieterich et al., 2018; Rinninella et al., 2018). However, the human salivary microbiota can include up to 700 bacterial species and, actually, it is quantitatively relevant (unlike esophagus and stomach), since it can reach a cell density of 10^11^ CFU/g in the dental plaque and 10^8–9^ in the saliva (Maukonen et al., 2008).

Even though most studies investigating the microbiome in CD patients were focused on duodenal and fecal samples, in the last few years the salivary microbiome has started to attract the interest of several researchers in this field, due to several reasons, such as its potential role in the disease pathogenesis, its correlation with the duodenal microbiota and, thus, a possible source of non-invasive biological markers (De Angelis et al., 2016).

In this mini-review, we summarize and discuss the available evidence regarding the composition of the oral microbiome in patients with CD, starting from its implication in the disease pathogenesis to arrive at the clinical studies in children and adults.

## Healthy Salivary Microbiota

The oral cavity is composed of different microbiological habitats, including several mucosal surfaces (e.g. gingiva, tongue, hard/soft cleft, lips/cheeks) and teeth, which are characterized by distinct mechanical and adhesive properties, as well as variable exposure to salivary secretion and its related chemical, enzymatic, and immunological actions. Therefore, all these sites inside the oral cavity are colonized by distinct microbial communities that overall form the salivary or oral microbiota (Dewhirst et al., 2010; Faran Ali and Tanwir, 2012).

Compared to the other upper GI tracts, the oral cavity is densely populated by microbes: indeed, one milliliter of saliva can contain approximately 10^8^ microbial cells (Maukonen et al., 2008; Willis and Gabaldón, 2020). In general, up to 700–1,000 microbiological species (including fungi and viruses) can be isolated from the oral cavity of human beings. Of course, bacterial flora is by far the main component of oral microbiota and includes *Firmicutes* spp., *Bacillus* spp., *Proteobacteria*, and *Actinomycetes* (Lu et al., 2019). However, each microenvironmental niche in the oral cavity contains a limited set of bacterial species. In general, facultative anaerobes, such as streptococci and *Actinomyces* species, represent the main component of the salivary microbiota, whereas in the subgingival area (whereby there is reduced oxygen tension) strict anaerobic bacteria, such as *Bacteroidaceae* spp. and spirochaetes, are more abundant (Lamont et al., 2018). Overall, a healthy individual microbiome usually comprises a range of 100 to 200 distinct bacterial species (Paster et al., 2006; Willis and Gabaldón, 2020).

Rather than describing the complexity of the inter-individual variations of salivary microbiome in health and disease (which is beyond our purposes in this mini-review), it is important to emphasize that the oral microbiota is a very dynamic entity, which profoundly changes during the life of an individual. Indeed, after the eruption of teeth, these hard surfaces enable much larger masses of microorganisms to accumulate as biofilms, in the dental plaque and in the subgingival spaces. Over time, dental procedures and the placement of orthodontics devices may further change the characteristics of oral microbiome, in addition to dietary factors, individual salivary properties, and pharmacological therapies (first of all, antibiotics), of course (Faran Ali and Tanwir, 2012).

Finally, it is worth to mention some initial and recent evidence supporting the influence of oral microbiome on the gut microbiota: indeed, salivary bacteria are continuously swallowed, can reach the gut and, thus, may colonize, persist and influence the local microbiota (du Teil Espina et al., 2019; Lu et al., 2019).

## Salivary Microbiota and Celiac Disease Pathogenesis

As mentioned, several animal and human studies currently support the implication of the GI microbiota in the pathogenesis of CD (Collado et al., 2007; Nadal et al., 2007; Schippa et al., 2010; Lindfors et al., 2019). Some murine models of CD-like enteropathy triggered by gluten showed that the microbiological environment of the intestinal lumen can play a crucial role in gluten-induced enteropathy and some probiotics can influence this pathological occurrence (D’Arienzo et al., 2011; Laparra et al., 2012; Papista et al., 2012). Even though a clear celiac microbiome signature is not evident in humans so far, several studies suggested that gut microbiota may influence the disease onset and progression by several mechanisms, such as the modulation of innate/adaptive immune system and the epithelial barrier permeability/reactivity (Verdu et al., 2015). Moreover, an impact on gluten peptides immunogenicity (e.g. *Rothia aeria*, *Pseudomonas aeruginosa*, *Lactobacillus* spp.) and/or the provision of inflammatory signals (e.g. *Neisseria flavescens*) was suggested for a number of specific bacterial species (Shan et al., 2002; D’Argenio et al., 2016; Caminero et al., 2016; Wei et al., 2020).

Several clinical and epidemiological studies confirmed the prominent role of class II MHC β chains and, particularly, that codified by DQB1*02 allele, in the immunological pathogenesis of CD, as summarized and highlighted by several systematic reviews and meta-analyses performed by our research group (De Silvestri et al., 2018; Capittini et al., 2019; Poddighe et al., 2020). Nenna et al. demonstrated that anti-transglutaminase (anti-tTG) autoantibody titers were significantly different in CD patients receiving a gluten-containing diet, according to the HLA-DQB1*02 status, in addition to having more expressed clinical and histopathological characteristics (Nenna et al., 2008). Molecular studies suggested that the high content of proline and glutamine residues of HLA-DQ2-restricted gliadin epitopes, is an important aspect to explain their interaction with class II MHC molecules (Sollid, 2002). A research based on X-ray crystallography proposed that some DQ2 β chain residues (in detail, Arg-β70 and Lys-β71 of β chains encoded by HLA-DQB1*02) can effectively interact with these residues of the gliadin epitopes (Kim et al., 1999). Importantly, recent studies also suggested that the HLA-DQ2 genotype can influence the early gut microbiota composition and, in detail, the presence of “pathogenic bacteria” that may promote a pro-inflammatory immunological environment in the gut, which could be favorable to CD development (Olivares et al., 2015; Olivares et al., 2018b). However, no studies have explored any potential relationship between HLA-DQ genes and the composition of salivary microbiome so far.

As regards the implication of salivary microbiota in the pathogenesis of CD, the modification of immunologically important gliadin peptides by enzymes produced by the oral bacteria is supposed to be the most likely mechanism modulating the CD risk in HLA-DQ genetically predisposed and gluten-exposed individuals. Glutenins and gliadins containing these peptic sequences rich of glutamine and proline residues, allow certain gluten-derived domains to be highly resistant to the degradation by human gastrointestinal proteases, including pepsin, trypsin, chymotrypsin, carboxypeptidases A and B, elastases, and intestinal brush-border membrane enzymes, in addition to conferring some immunological peculiarities that stimulate CD4+ T cells in the lamina propria of HLA-DQ2-positive patients with CD (Vader et al., 2002; Wieser, 2007). In detail, Fernandez Feo et al. analyzed the human oral microbiome for bacteria with proteolytic enzymes that are able to cleave intact gliadins and derived immunodominant peptides, in order to investigate whether some bacteria may play any physiological roles in the digestion of gluten. Interestingly, they identified gluten-degrading microorganisms in dental plaque and saliva, which can affect the digestion of the immunogenic gliadins implicated in CD immune-pathogenesis (Fernandez-Feo et al., 2013).

Eventually, Tian et al. supported this pathogenic hypothesis by demonstrating that the hydrolysis of gluten substrates (including the alpha-gliadin-derived immunogenic 33-mer peptide) in human saliva was significantly higher in CD patients compared to the controls. Interestingly, these differences persisted even after the normalization for total protein or total bacterial load. Therefore, these authors suggested that some oral microbe-derived enzyme activities, which resulted to be more pronounced in CD patients, may finally affect the gluten-derived peptides immunological processing and, in detail, the antigenic presentation of immunogenic gluten epitopes at the level of intestinal immune system (Tian et al., 2017).

This general perspective that the proteolytic activities of the gastrointestinal bacteria (including those colonizing the oropharyngeal tract) may affect the individual immunoreactivity to gluten, can be supported by the recent study by Caminero et al.: here, mice at genetic risk for CD resulted to be more prone to develop the disease when gluten exposure was associated with *P. aeruginosa* elastases. Moreover, the authors clinically translated this concept by showing that duodenal biopsies from patients with active CD presented an increased proteolytic activity against gluten substrates, which correlates with a greater abundance of Proteobacteria, including *Pseudomonas* spp. (Caminero et al., 2019).

## Salivary Microbiome in Celiac Children

Acar et al. first showed that the salivary microbiota of CD children (n = 35) can significantly differ from healthy controls (n = 35), despite the adherence to the gluten-free diet. In detail, they found that the prevalence of salivary “*mutans*” streptococci and lactobacilli colonization was significantly lower in the CD group. Interestingly, they also reported a significantly higher prevalence of enamel defects and recurrent aphthous oral manifestations (which are well-known CD clinical features) in these CD children (Acar et al., 2012).

Francavilla et al. investigated the salivary microbiota in children affected with CD. By investigating CD pediatric patients (n = 13) on gluten-free diet (for at least 2 years) and as many age-matched healthy controls, they reported some significant differences in terms of microbiota composition and metabolic characteristics. By culture-based methods, the number of total anaerobes resulted to be significantly decreased in the saliva of CD children and, in terms of other bacterial species, a significant difference was found only in the number of *Enterobacteriaceae*, which was lower in controls. However, the 16S rRNA gene analysis of microbial diversity showed several differences between the study groups in the ratios of some *Firmicutes* and *Actinobacteria* to *Bacteroidetes*. Importantly, this study emphasized that the oral microbiome is not restored by the gluten-free diet and, thus, some microbial parameters and metabolomic features may represent signatures of CD patients (Francavilla et al., 2014).

However, another interesting study by the same group of researchers actually showed that the dietary style and changes are able to significantly affect the microbiome and metabolomic characteristics of the oral environment of treated CD children. Indeed, they investigated a group of CD children (n = 14) of Saharawi ethnicity (known to have a high prevalence of CD) following an African-style gluten-free diet for at least 2 years, before and 60 days after the beginning of an Italian-style gluten-free diet. After the implementation of an Italian-style gluten-free diet, significant differences in the abundance of several bacterial species were noticed, showing that the individual “salivary biotype” had changed. Accordingly, the metabolic profile of the microbiota changed as well, showing several metabolic differences for amino acids, vitamins, and co-factors (Ercolini et al., 2015).

From one side this study emphasized the profound influence of diet factors other than gluten on the salivary microbiome, making it more difficult to describe a CD signature; on the other side, it might suggest how these additional dietary aspects could affect the risk of developing this disease, considering that the allelic frequency of the HLA-DQ predisposing genetic background in Saharawi population is not much higher than in Caucasians (Catassi et al., 2001). Unfortunately, there are no study comparing the salivary microbiota of CD children on gluten free diet with children at the diagnosis, thus, with active CD.

A schematic and chronological overview summarizing the main methodological aspects and microbiological findings of the available studies on salivary microbiome in pediatric and adult CD, is provided in **Table 1**.

**Table 1:** Schematic and chronological overview of the clinical studies assessing the salivary microbiome in CD patients.

Authorship(country, year)	Study population	CD patients	CD stage	Controls (number)	Study objective	Main microbiological conclusions
**Acar et al.** **(Turkey,** **2012** **)**	Children Age range: 6–19 yrs.	35	T-CD*	HC (n = 35)	“to investigate the prevalence of dental enamel defects, recurrent aphthous stomatitis, and caries experience, and to measure salivary flow rate, buffer capacity, saliva and plaque pH, and salivary cariogenic salivary cariogenic microflora in CD and in healthy subjects.”	“CD appeared to be associated with a significantly higher prevalence of developing enamel defects and RAS, but a lower prevalence of salivary *mutans* streptococci and lactobacilli colonization,…”
**Francavilla et al.** **(Italy,** **2014****)**	Children Median age: 10 ± 1.4 yrs.	13	T-CD*	HC (n = 13)	“to compare the salivary microbiota and metabolomes of CD children subjected to GFD and healthy children.”	“Some microbial indices (e.g., ratios of some Firmicutes and Actinobacteria to Bacteroidetes) and the levels of some metabolites (e.g., nonanal, ethyl-acetate, and 2-exanone) are signatures of CD patients. GFD lasting at least 2 years did not completely restore the salivary microbiota and, consequently, the salivary metabolome of T-CD children.”
**Ercolini et al.** **(Italy,** **2015****)**	Children Median age: 8.4 ± 0.7 yrs.	14	T-CD	N/A	To investigate “the salivary microbiota and metabolome of Saharawi celiac children in response to the change from the traditional African- to the Italian-style gluten-free diet.”	“Significant differences were identified in the salivary microbiota and metabolome when Saharawi celiac children switched from African- to Italian-style dietary habits. An Italian-style gluten-free diet caused increases in the abundance of *Granulicatella*, *Porphyromonas* and *Neisseria* and decreases in *Clostridium*, *Prevotella* and *Veillonella*, altering the ‘salivary type’ of the individuals.”
**Tian et al.** **(USA,** **2017****)**	Adults Age range: 18–71 yrs.	29	T-CD* (n = 21) RCD (n = 8)	HC (n = 20) FGID (n = 12)	To “investigate salivary enzymatic activities and oral microbial profiles in healthy subjects versus patients with classical and refractory CD.”	“Salivary glutenase activities were higher in CD patients compared to controls, both before and after normalization for protein concentration or bacterial load. The oral microbiomes of CD and RCD patients showed significant differences from that of healthy subjects, e.g., higher salivary levels of lactobacilli (P < 0.05), which may partly explain the observed higher gluten-degrading activities.”
**Iaffaldano et al.** **(Italy,** **2018****)**	Adults Age range^#^: >18 yrs.	36	a-CD (n = 14) T-CD* (n = 22)	HC (n = 20)	To “investigate the oropharyngeal microbiome in CD patients and controls to evaluate whether this niche share microbial composition with the duodenum.”	“*Bacteroidetes*, *Proteobacteria* and *Firmicutes* differed significantly between the three groups. In particular, *Proteobacteria* abounded in a-CD and *Neisseria* species mostly accounted for this abundance (p<0.001), whereas *Bacteroidetes* were more present in control and GFD microbiomes. Culture-based oropharyngeal microbiota analysis confirmed the greater abundance of *Proteobacteria* and of *Neisseria* species in a-CD.”
**Panelli et al.** **(Italy,** **2020****)**	Adults Age range^$^: >18 yrs.	52	a-CD (n = 13) T-CD (n = 29) RCD (n = 4) p-CD (n = 6)	FGID (n = 31)	“To determine the microbiota structure and composition of salivary, duodenal mucosa, and stool samples, followed by appropriate bioinformatic analyses.”	“…a critical decrease of *Firmicutes* and *Actinobacteria* and an expansion of *Proteobacteria* at mucosal and salivary levels appear evident in all CD groups, mostly in a-CD and RCD, in comparison with the C group that does not normalize in T-CD. By contrast, no clear differences are evident at the stool level probably due to the high variability among samples. As regards the relative distribution of genera, an expansion of Neisseria and a reduction of *Streptococcus* in CD groups with respect to the C group emerge in both mucosal and salivary communities, whereas *Bacteroides* is the predominant one predominant in the stool consortium.”

CD, Celiac Disease; T-CD, treated CD in remission; a-CD, active CD; RCD, refractory CD; p-CD, potential CD; HC, healthy controls; FGID, functional gastrointestinal disease; N/A, not available; n, number; yrs., years; GFD, gluten free diet.

*The authors specify that CD patients were on gluten-free diet for 2 years at the time of the study enrollment.

^Even though this study is authored by Italian researchers, the study population includes only Saharawi children.

^#^Median age ( ± SEM): Controls: 30 ± 0.4 yrs.; T-CD: 32 ± 0.4 yrs.; a-CD: 26 ± 0.4 yrs.

^$^Median age ( ± SD): p-CD: 41 ± 14 yrs.; a-CD: 35 ± 6 yrs.; T-CD: 37 ± 6 yrs.; RCD: 53 ± 15 yrs.; Controls: 44 ± 17 yrs.

## Salivary Microbiome in Celiac Adults

The previously mentioned study on salivary enzymatic activities by Tian et al. also provided some data about the local microbiome in adult CD patients. Indeed, they analyzed the saliva from patients with treated CD in remission (n = 21) and refractory CD (n = 8), in addition to healthy controls (n = 20) and individuals with functional gastrointestinal complaints (n = 12). They described some significant differences in the oral microbiome of CD patients compared to the healthy subjects and, in detail, higher salivary levels of lactobacilli were found in the former group, which may partially correlate with the observed higher gluten-degrading activities. Interestingly, some significant differences in bacterial phyla and species composition were also reported between CD patients in remission and patients with refractory CD. Indeed, Bacteroidetes and Fusobacteria were significantly more abundant in CD patients in remission than in the refractory CD group, where actually Actinobacteria population was more consistent. Accordingly, 22 species (many belonging to Firmicutes phylum as well) were more numerous in gluten-free diet responding CD patients than in refractory CD patients, in whom some bacterial species (most of them belonging to the Actinobacteria phylum) were comparatively more represented (Tian et al., 2017).

Iaffaldano et al. characterized by 16S rRNA sequencing and by culture-based methods the oropharyngeal microbiota in active CD patients (n = 14), CD patients treated with gluten-free diet (n = 22), and control subjects (n = 20). Both analytical approaches showed that Proteobacteria were significantly more abundant in untreated CD patients, whereas Bacteroidetes were prevalent in controls and treated CD patients. Additionally, the authors reported that differences in *Neisseria* spp. abundance mostly accounted for the greater abundance of Proteobacteria in the microbiota of the active CD group. In this regard, the authors also suggested some similarities between the oropharyngeal and duodenal microbiomes in CD patients (Iaffaldano et al., 2018). Following these initial findings, the same authors recently published a study in which they set up and performed a fast qPCR-based method to further investigate the abundance of the CD-associated *Neisseria* spp. in the oropharynx of both active (n = 14) and gluten-free diet CD patients (n = 22), in addition to a control group (n = 20). Here, they confirmed that *Neisseria* spp. were significantly more abundant in active CD patients than in the other two groups, which did not show any significant differences between themselves (Esposito et al., 2019).

Very recently, Panelli et al. investigated 52 adult patients affected with CD (including: active CD, n = 13; treated CD, n = 29; refractory CD, n = 4; potential CD, n = 6) and 31 patients with functional dyspepsia, in order to characterize the salivary, duodenal, and fecal microbiota composition. In addition to a general reduction of the microbial diversity in all analyzed samples from CD patients (ranging from mild reduction in potential CD and the nadir achieved in refractory CD), this study showed a significant abundance of Proteobacteria in active CD and, importantly, confirmed the expansion of *Neisseria* spp.; moreover, they reported a better correspondence of the bacterial microbiota in the saliva with duodenal mucosa microbiome, rather than with fecal samples (Panelli et al., 2020).

## Conclusion

The clinical studies investigating the salivary microbiome in CD children and adults are definitely less numerous than those performed on duodenal and fecal microbiome. Interestingly, most of the available studies on oral microbiota have been performed in Italy and, thus, mainly describe the Italian population. Compared to fecal microbiota, salivary microbiome could correlate better with the duodenal bacterial environment, where the main immuno-pathological events leading to the development of CD, take place.

Therefore, further clinical studies on salivary microbiome by different and independent research groups and in different populations, are advisable in order to explore the usefulness of the salivary analysis and better understand some aspects of CD pathogenesis with potential clinical and practical implications.

## Author Contributions

DP conceived and wrote the manuscript. AK provided expert and intellectual contribution. All authors contributed to the article and approved the submitted version.

## Funding

This review was supported by the Nazarbayev University Faculty Development Competitive Research Grant 2020-2022, No. 240919FD3912, and the Nazarbayev University Social Policy Grant.

## Conflict of Interest

The authors declare that the research was conducted in the absence of any commercial or financial relationships that could be construed as a potential conflict of interest.
